# The pyroptosis-related gene signature predicts prognosis and reveals immune microenvironment infiltration in reclassified glioblastoma based on 2021 WHO classification

**DOI:** 10.3389/fimmu.2025.1617036

**Published:** 2025-07-21

**Authors:** Xiaohong Zheng, Zhangchi Pan, Lina Chen, Can Wang, Wenbin Li

**Affiliations:** ^1^ Department of Medical Oncology, Fujian Medical University Union Hospital, Fuzhou, Fujian, China; ^2^ Department of Neurology, Fujian Medical University Union Hospital, Fuzhou, Fujian, China; ^3^ Department of Neuro-Oncology, Cancer Center, Beijing Tiantan Hospital, Capital Medical University, Beijing, China; ^4^ Hepato-Pancreato-Biliary Center, School of Clinical Medicine, Beijing Tsinghua Changgung Hospital, Tsinghua University, Beijing, China

**Keywords:** pyroptosis, glioblastoma, immune infiltration, clinical therapy, prognostic model

## Abstract

**Background:**

Glioblastoma (GBM) is a highly malignant brain tumor with a poor prognosis. The WHO Classification of Tumors of the Central Nervous System (WHO CNS5) reclassified GBM in 2021. Pyroptosis, as an inflammatory form of programmed cell death, could regulate tumor cell proliferation, invasion, and metastasis. However, the role of pyroptosis in the newly defined GBM and its correlation with immunity have not yet been elucidated.

**Method:**

According to the 2021 WHO CNS5, a total of 209 newly defined GBM samples from The Cancer Genome Atlas (TCGA) cohort were included for analysis. The Chinese Glioma Genome Atlas (CGGA) cohort was used as the validation set. The prognosis model was built by Least Absolute Shrinkage and Selection Operator (LASSO) Cox analysis. The nomogram was conducted to confirm the prognostic value of risk score. ESTIMATE, CIBERSORTx, and ImmuCellAI were used to investigate immune infiltration. Quantitative real-time polymerase chain reaction (RT-qPCR) and immunohistochemistry were performed to validate PLCG1 and NOD2 genes in the prognostic model.

**Results:**

The risk score model including the two genes was built. The experimental results verified that elevated NOD2 expression and reduced PLCG1 expression in GBM represent poor prognosis with a higher risk. This risk score model could predict the survival rates of patients with GBM with medium to high accuracy. The benefit of chemotherapy or radiotherapy was greater in the high-risk group than in the low-risk group. Moreover, the high-risk group had stronger immune activity and poorer immunotherapy response.

**Conclusions:**

In summary, our study provided strong evidence for the prognosis and clinical management of the newly defined GBM from bioinformatics and experimental analysis. Furthermore, our findings provided a foundation for future research targeting pyroptosis and its immune microenvironment to improve GBM prognosis.

## Introduction

Glioblastoma (GBM) is one of the most aggressive and deadliest central nervous system tumors. GBM has a dismal prognosis, indicated by its particularly low survival rate of 7.2% in 5 years (1). The current main treatments for GBM are surgical resection followed by adjuvant chemoradiotherapy. Despite the tremendous effort that has been devoted to develop novel therapies, the 5-year survival rate has been slow to improve. Given the limitations of GBM treatments, new therapeutic targets are needed to improve the survival rate of GBM. Therefore, reliable novel prognostic models are urgently required to make targeted therapies more feasible.

Although many biomarkers or gene signatures have been found to have the potential to predict the prognosis of GBM, they are still in the molecular research phase and have not yet been applied in clinical practice. Moreover, in 2021, the fifth edition of the WHO Classification of Tumors of the Central Nervous System (WHO CNS5) reclassified GBM—removing the isocitrate dehydrogenase (IDH) mutation type from GBM and adding the IDH-wild-type diffuse astrocytoma in adults with TERT promoter mutation or EGFR gene amplification or +7/−10 chromosome copy number changes (2). This significant change will affect the survival time range of GBM. Thus, revealing prognostic gene signatures for the prognosis of the newly defined GBM would be of great significance.

It is known that there are several types of cell death in cancer treatment, including necrosis, apoptosis, necroptosis, autophagy, and pyroptosis. Pyroptosis is considered to be a novel inflammatory form of programmed cell death triggered by certain inflammasomes (3). Pyroptosis relies on the cleavage of gasdermins via classical and non-classical pathways and can lead to the continuous expansion of cells until the cell membrane ruptures and causes the cell content to flow out, thus triggering a strong inflammatory response (4, 5). According to previous studies, pyroptosis primarily exists to defend against intracellular infection and trigger an inflammatory anti-microbial response through the release of damage-associated molecular patterns (6). An increasing number of studies suggest that pyroptosis plays an important role in the prognosis of cancer (7, 8). It has been reported that inflammatory vesicles, gasdermin proteins, and proinflammatory cytokines, which are key components of pyroptosis, are associated with tumor cell proliferation, invasion, and metastasis (9). Given that pyroptosis plays a key role in the development of tumors and antitumor processes, some recent studies identified a novel pyroptosis-related gene signature for the prognosis of ovarian cancer, lung adenocarcinoma, and gastric cancer (10–12). The prognostic value of pyroptosis-related genes in the newly defined GBM has not yet been elucidated.

Because of the limited prognostic markers of GBM in clinical practice and the limitations of GBM treatment, there is an urgent need for the development of an effective gene signature to indicate prognosis and guide clinical treatment. Thus, we performed a systematic study to determine the expression levels of pyroptosis-related genes in the newly defined GBM, investigate the prognostic value of these genes, explore whether prognostic model based on these genes can guide the clinical therapeutics of GBM, and study the correlations between the prognostic model of pyroptosis-related genes and the tumor immune microenvironment.

## Materials and methods

### Sources of glioblastoma datasets and preprocessing

The RNA sequencing (RNA-seq) data and the corresponding clinical information of all patients with glioma (including low-grade glioma and GBM) and somatic datasets were obtained from The Cancer Genome Atlas (TCGA) database on 18 August 2021. The copy number variation (CNV) data and the single-nucleotide polymorphism (SNP) data of all glioma were downloaded from the University of California, Santa Cruz (UCSC) Xena website. Then, according to the new classification of GBM based on the 2021 WHO CNS5, we included adult GBM that were IDH-wildtype and adult diffuse astrocytic tumors that were IDH-wildtype with TERT promoter mutation or EGFR gene amplification or +7/−10 chromosome copy number alterations (2). EGFR was said to be amplified if the respective probes exhibited an intensity higher than 0.6 on a log2 scale (13). The records of duplicate patients were removed. Finally, the datasets of 209 patients with the newly defined GBM were included for further analysis. The validation sets were downloaded from the Chinese Glioma Genome Atlas (CGGA) database. The IDH-mutation GBM and lower-grade glioma (LGG) were removed from the CGGA325 set. We excluded pediatric patients from both the TCGA and CGGA sets. Before model fitting, the gene expression data from the TCGA cohort and CGGA cohort were processed by log2 transformation [log2(FPKM+1)]. Fragment per kilobase million (FPKM) data were used for single-sample gene set enrichment analysis (ssGSEA) and transcripts per kilobase million (TPM) data were used for CIBERSORTx and ImmuCellAI analysis.

### Defining pyroptosis-related genes

We extracted 33 pyroptosis-related genes from prior reviews (10, 14–17), and they are presented in **Supplementary Excel File 1**.

### Mutation analysis of pyroptosis-related genes

The mutation frequency and oncoplot waterfall plot of 33 pyroptosis-related genes in patients with GBM were generated based on the SNP data. The location of CNV alteration of 33 pyroptosis-related genes on 23 chromosomes and the CNV variation frequency of these genes were drawn based on the CNV data.

### Construction and validation of the prognostic model of pyroptosis-related genes

To develop a robust prognostic signature based on pyroptosis-related genes, we employed a multi-step feature selection strategy: First, using Kaplan–Meier (KM) survival analysis with Log-rank testing, we evaluated the individual prognostic significance of all 33 pyroptosis-related genes. Genes significantly associated with overall survival (OS) (Log-rank *p* < 0.05) were retained for further model building. This step yielded eight candidate genes: AIM2, CASP4, IL1B, NLRC4, NOD2, PLCG1, PYCARD, and SCAF11. To reduce dimensionality, mitigate potential overfitting, and address multicollinearity among the eight candidate genes, we applied the Least Absolute Shrinkage and Selection Operator (LASSO) Cox regression analysis. This was performed using the R package glmnet. Crucially, to enhance the stability and reliability of the feature selection, we implemented 1,000 iterations of 10-fold cross-validation during the LASSO regression process. This rigorous approach resulted in the retention of six genes: AIM2, CASP4, IL1B, NOD2, PLCG1, and SCAF11. Finally, to refine the model and identify the most parsimonious set of genes with optimal prognostic power, we performed bidirectional stepwise Cox proportional hazards regression based on the Akaike Information Criterion (AIC) using the R package MASS. This stepwise selection process, guided by AIC minimization, identified the two most prognostically informative core genes: NOD2 and PLCG1. These two genes formed the basis of our final prognostic risk score model. The risk score formula was as follows: risk score = 2.74415*e^(0.4763*NOD2-0.3042*PLCG1)^. The cutoff point was determined using the “survminer” package. The patients with GBM were divided into low- and high-risk subgroups according to the best cutoff value of risk score. We used KM survival curves to compare the OS between the two subgroups and time-dependent receiver operating characteristic (ROC) curves to determine the efficiency of the model. For the validation studies, the GBM cohort from the CGGA database was employed. The risk score was calculated by the same formula used for the TCGA cohort. By applying the best cutoff value risk score from the TCGA cohort, the patients in the CGGA cohort were also divided into low- or high-risk subgroups, and these groups were then compared to validate the gene model.

### Independent prognostic analysis of the risk model

We extracted the clinical information (gender, radiotherapy, chemotherapy, MGMT promoter, subtype, and age) of patients in the GBM datasets. These variables were analyzed in combination with the risk score in our regression model. Univariate and multivariate Cox regression models were employed for the analysis. A forest was used to show the *p*-value, HR, and 95% CI of each variable through the “forestplot” R package.

### Development of the nomogram

A nomogram to predict the 6-, 12-, and 24-month survival probability was developed according to the results of multivariate Cox stepwise regression analysis. The bootstrap sampling method and the construction of a calibration curve were used to evaluate the effect of the nomogram. The bootstrap-C-index and bootstrap-Brier Score were used to assess the consistency between the model prediction results and the actual observation results.

### Drug sensitivity and treatment subgroup analysis based on the risk score model

In treatment subgroup analysis, according to the risk score model, we used KM survival curves to compare the progression-free survival (PFS) and OS in different treatment subgroups. Based on drug response data from the Cancer Therapeutics Response Portal (CTRP) and Cancer Cell Line Encyclopedia (CCLE), we screened drugs whose response was correlated with pyroptosis-related genes in GBM using Spearman’s correlation analysis. The drugs and genes with an absolute value of correlation coefficient >0.3 (18) and *p* < 0.05 were considered statistically correlated. Positive correlation indicated that the higher the gene expression level, the higher the area under the concentration–response curve (AUC) value of sensitivity scores and the lower the sensitivity to drugs, and *vice versa*.

### Immune infiltration, tumor mutation burden, and microsatellite instability analysis

In the GBM microenvironment, immune and stromal cells are two key types of nontumor components and have been indicated to be significant for the diagnosis and prognosis of tumors. Yoshihara et al. (19) designed the ESTIMATE algorithm to compute immune and stromal cell scores to predict the infiltration of these nontumor cells. We used ESTIMATE to evaluate immune scores, ESTIMATE scores, stromal scores, and tumor purity scores in each tumor sample and to determine the immune infiltration level in combination with the risk score.

ssGSEA, which assisted in quantifying the enrichment level of an immune cell/signature, pathway, or biological process in a tumor sample, was used to assess the gene score of every gene set for every sample (20). The infiltration of immune cells in the microenvironment was determined by 29 immune pathways, and GBM samples were hierarchically clustered into “immune-high (immune-H)” and “immune-low (immune-L)”.

We used CIBERSORTx and ImmuCellAI (21) to quantify the proportions of immune cells. CIBERSORTx is an analytical tool used to impute gene expression profiles and provide an estimation of the abundances of member cell types in a mixed cell population, using gene expression data. ImmuCellAI is a web-based analytical and discovery platform for analyzing the abundance of 24 immune cells from the gene expression dataset. Moreover, ImmuCellAI can be applied to estimate the difference of immune cell infiltration in diverse groups as well as predict patient response to immune checkpoint blockade therapy. We selected immune cells associated with risk score (Spearman correlation coefficient |cor| > 0.3).

In tumor mutation burden (TMB) and microsatellite instability (MSI) analysis, Spearman’s correlation analysis was performed to calculate the correlation between gene expression and TMB and MSI score. Spearman correlation coefficient |*R*| > 0.3 and a *p*-value of < 0.05 were considered statistically significant.

### Quantitative real-time polymerase chain reaction

To validate the PLCG1 and NOD2 of the prognostic model, tumor tissue was collected from 12 patients with glioma from Beijing Tiantan Hospital, Capital Medical University. Among them, six cases of gliomas (including one WHO grade 2 astrocytoma, one WHO grade 3 astrocytoma, and four GBMs) were used for quantitative real-time polymerase chain reaction (RT-qPCR), and six cases of GBM were used for immunohistochemistry.

mRNA expressions were detected using RT-qPCR assay. Briefly, total RNA was extracted from the glioma samples using the Tissue RNA Purification Kit Plus (ESscience Biotech) and reversely transcribed to cDNA via the PrimerScript RT reagent Kit with gDNA Eraser (Takara). Quantitative PCR was performed using PowerUp™ SYBR™ Green Master Mix (Thermo Fisher). The primers used in this study were obtained from Sangon Biotech (Shanghai, China), including PLCG1 (Forward 5′-GGATCAAGGGCTTAACTTGGC-3′, Reverse 5′-GACCCGGTAGTTGACCTGG-3′), NOD2 (Forward 5′-CACCGTCTGGAATAAGGGTACT-3′, Reverse 5′-TTCATACTGGCTGACGAAACC-3′), and β-actin (Forward: 5′-CATTCCAAATATGAGATGCGTTGT-3′, Reverse: 5′-TGTGGACTTGGGAGAGGACT-3′). The relative mRNA levels were calculated by the 2−^δδCt^ method.

### Immunohistochemistry

PLCG1 and NOD2 staining was performed with formalin-fixed, paraffin-embedded GBM tissues. For PLCG1 and NOD2 staining, specimens from patients with rapidly progressing GBM and those with long-term stable GBM were used and samples from three individuals were collected in each group. The PFS of the six patients was as follows: P1: 26 months, P2: 13 months, P3: 16 months, P4: 4 months, P5: 6 months, and P6: 8 months. We defined patients with a PFS ≤ 12 months as those with rapidly progressing GBM and patients with a PFS >12 months as those with long-term stable GBM. All these patients with GBM met the diagnostic criteria for GBM according to the 2021 WHO CNS5. The slides were incubated with primary antibody (PLCG1: 1:100 dilution, Cell Signaling Technology; NOD2: 1:50, Affinity Biosciences) overnight at 4°C and then with horseradish peroxidase (HRP)-conjugated secondary antibody (ZSGB-Bio, Beijing, China) at room temperature for 1 h. DAB was used for staining. The intensity and density of the staining were examined by two investigators independently.

### Statistical analysis

**Figure 1:**
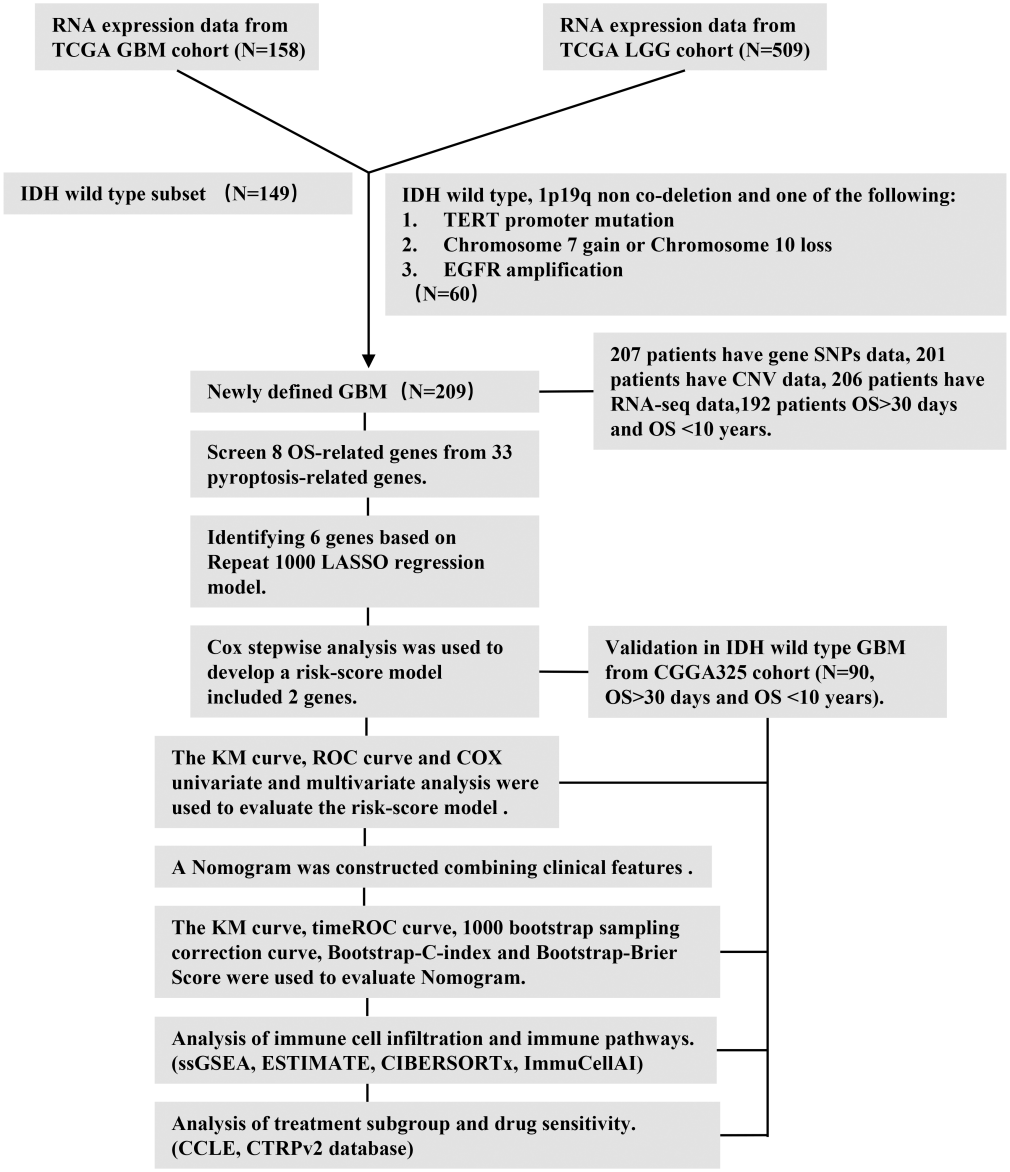
Workflow diagram. The specific workflow graph of data analysis.

The workflow chart (**Figure 1**) describes which samples were utilized at each stage of statistical analysis. All data analyses were performed with the R (version 4.1.1) and R Bioconductor packages. Detailed usage of the R packages is shown in the **Supplementary Text File**. A *p*-value of < 0.05 was considered to be statistically significant.

## Results

### Landscape of genetic variation of pyroptosis-related genes in the newly defined GBM

**Figure 2:**
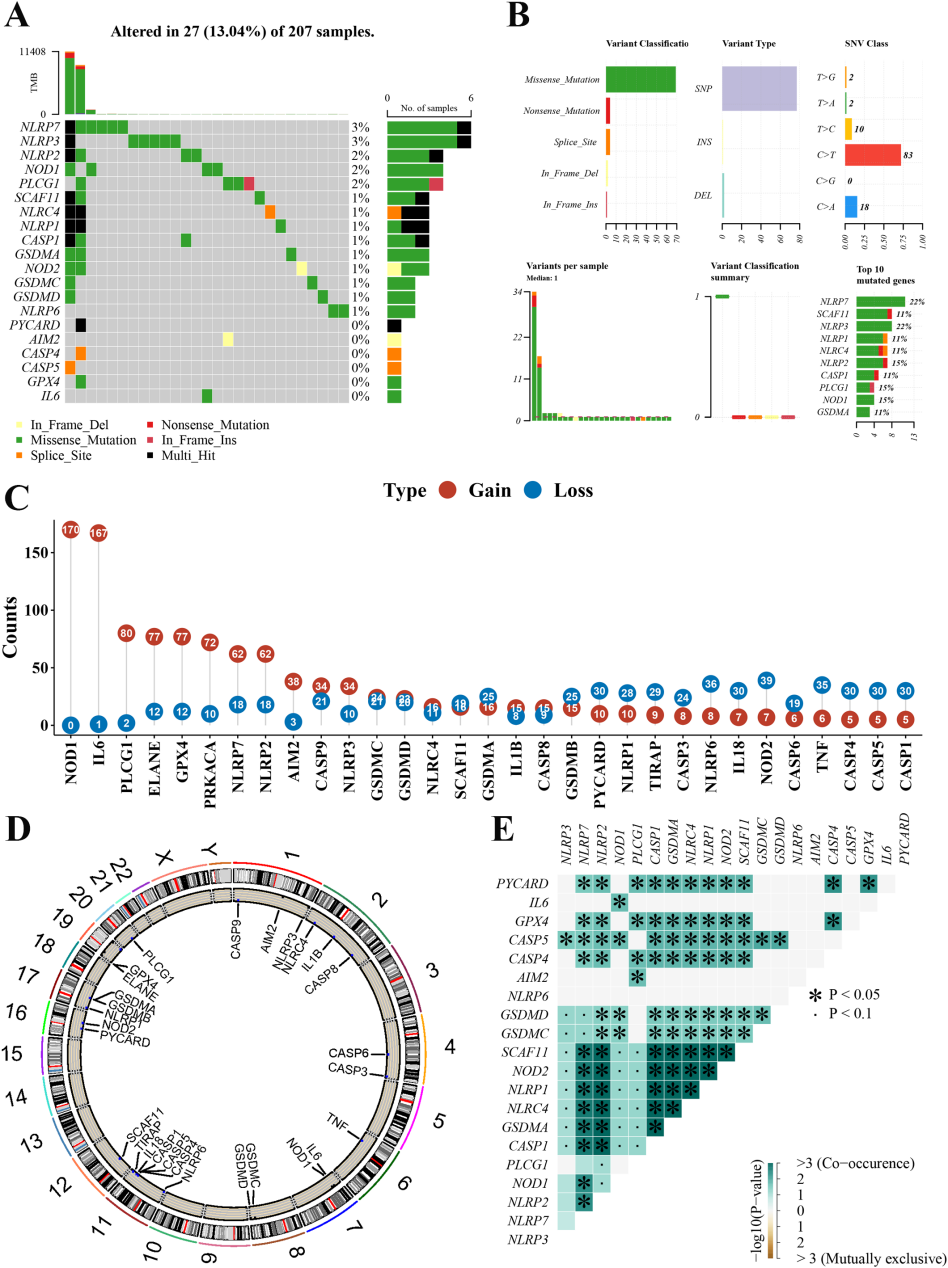
Landscape of genetic variation of pyroptosis-related genes in the newly defined GBM. **(A)** The mutation profiles in the 207 newly defined GBM. **(B)** The mutation frequency and classification of 20 pyroptosis-related genes in the GBM. **(C)** The CNV variation frequency of 31 pyroptosis-related genes in the GBM. The number represented the alteration counts. **(D)** The location of CNV alteration of 33 pyroptosis-related genes on 23 chromosomes in the GBM. **(E)** The mutation correlation between the 20 pyroptosis-related genes in the GBM. GBM, glioblastoma; CNV, copy number variation; SNP, single-nucleotide polymorphism; INS, insertion; DEL, deletion. *mean: *P<0.05, the pair of genes had significant mutational associations.

We summarized the incidence of CNV and somatic mutations of 33 pyroptosis-related genes in the newly defined GBM. As shown in **Figure 2A**, 27 of 207 (13.04%) GBM samples showed pyroptosis-related regulator mutations. Missense mutation was the most common variant classification (**Figure 2B**). SNPs were the most common variant type in missense mutation, and C > T ranked as the top single nucleotide variant (SNV) class. There were 20 mutations in the 33 pyroptosis-related genes, among which NLRP7 and NLRP3 showed the highest frequency of mutations (**Figure 2B**). We also explored CNV alteration frequency, which revealed that these genes showed prevalent CNV alterations. Among 33 pyroptosis-related genes, 2 genes did not have any gain or loss in any of the GBM samples. More than half of the 31 pyroptosis-related genes had copy number amplification, while the CNV deletion frequencies of GSDMA, GSDMB, PYCARD, NLRP1, TIRAP, CASP3, NLRP6, IL18, NOD2, CASP6, TNF, CASP4, CASP5, and CASP1 were widespread (**Figure 2C**). **Figure 2D** presents the location of CNV alterations of these 33 pyroptosis-related genes on chromosomes. Meanwhile, we analyzed the mutation correlation between the 20 pyroptosis-related genes (**Figure 2E**). The results demonstrated that 23 pairs of genes had significant mutational associations [−log10(*p*-value)>3 and *p* < 0.05].

### Construction of the pyroptosis-related prognostic gene model and validation using the CGGA database and experiments

The baseline characteristics of the newly defined GBM in the TCGA cohort and CGGA cohort are shown in **Supplementary Table 1**. A total of 209 newly defined GBM samples from the TCGA cohort were matched with the corresponding patients who had complete survival information. A total of 203 (patients with PFS > 5 days) and 192 (patients with OS > 30 days) patients with GBM were included in PFS and OS analysis, respectively.

**Figure 3:**
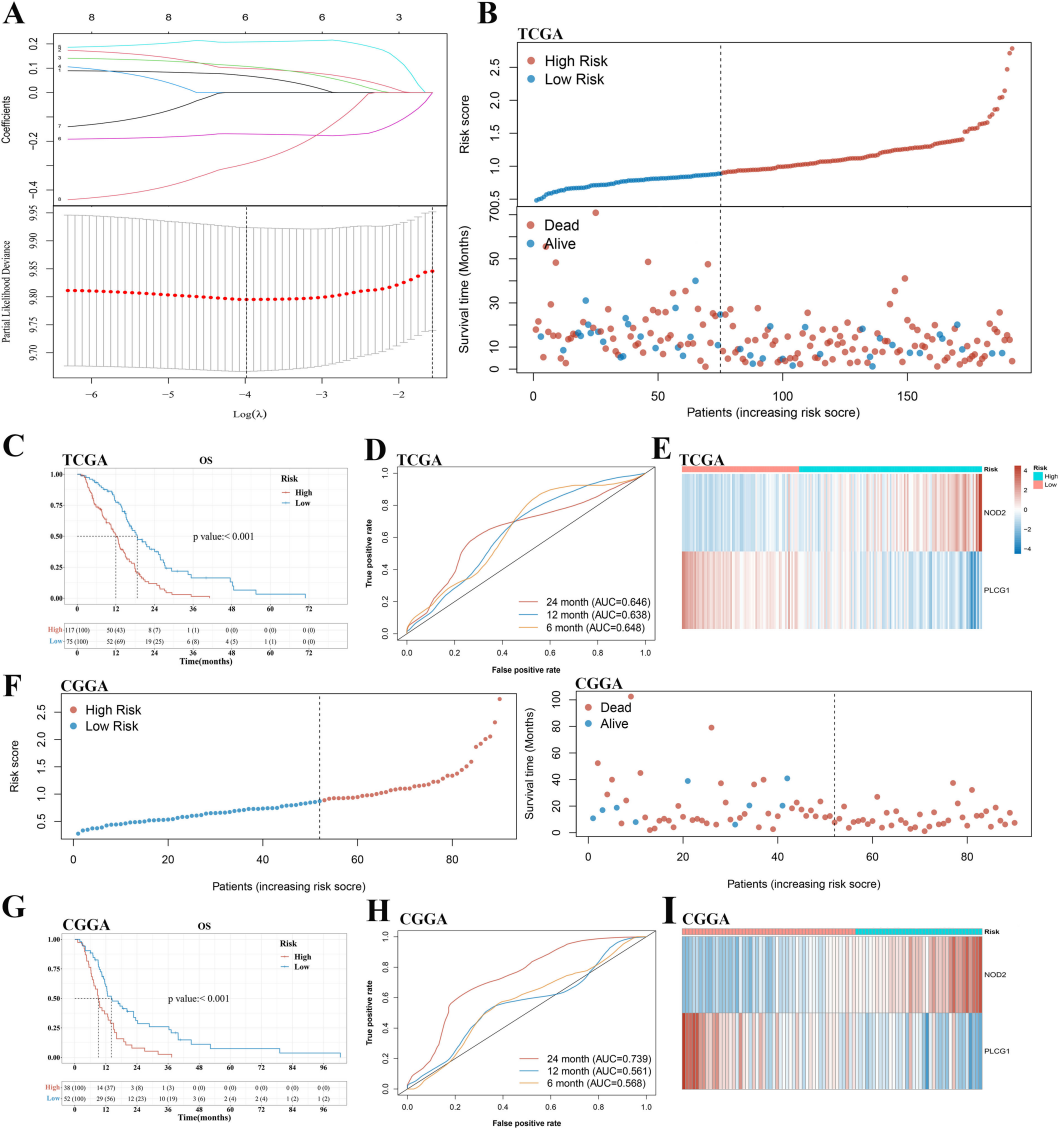
Construction and validation of a prognostic pyroptosis-related gene model in the TCGA cohort and CGGA cohort, respectively. **(A)** LASSO coefficient profiles of the eight pyroptosis-related genes and cross-validation for tuning the parameter selection in the LASSO regression. **(B, F)** Distribution of risk score and survival status of patients with GBM in the TCGA cohort and CGGA cohort, respectively. **(C, G)** Kaplan–Meier curves for the OS in the GBM high- and low-risk groups of the TCGA cohort and CGGA cohort, respectively. **(D, H)** Time-dependent ROC curves for GBM in the TCGA cohort and CGGA cohort, respectively. **(E, I)** The expression of PLCG1 and NOD2 of patients with GBM in the TCGA cohort and CGGA cohort, respectively. GBM, glioblastoma.

To develop a prognostic gene model, a KM analysis was performed on OS and PFS to screen those pyroptosis-related genes with a prognostic value. As a result, 8 and 10 genes with prognostic value were identified based on OS and PFS KM survival curves, respectively. The results suggested a poor OS in patients with GBM with low AIM2, CASP4, IL1B, NLRC4, NOD2, and PYCARD expression and high PLCG1 and SCAF11 expression (**Supplementary Figure 1A**), as well as a poor PFS with low AIM2, CASP4, GPX4, GSDMC, IL1B, NLRC4, NLRP3, NOD2, and PYCARD expression and high PLCG1 expression (**Supplementary Figure 1B**). LASSO Cox regression analysis was performed to construct a prognostic gene model based on these eight pyroptosis-related genes of OS (**Figure 3A**). Finally, two genes (PLCG1 and NOD2) were included in the risk score model by stepwise regression algorithm. The risk score = 2.74415*e^(0.4763*NOD2−0.3042*PLCG1)^. This risk score model had good universality (the related information is provided in **Supplementary Excel File 2**). Based on the risk score, patients with GBM were divided into a high- and a low-risk group with the best cutoff value of 0.89 (**Figure 3B**). As the risk score increased, the number of patients dying increased and survival decreased (**Figure 3B**). The KM curve demonstrated that the high-risk group had a shorter survival time than the low-risk group (**Figure 3C**). Time-dependent ROC analysis was applied to evaluate the sensitivity and specificity of the prognostic model, and we found the area under the ROC curve (AUC) to be 0.648, 0.638, and 0.646 for the 6-, 12-, and 24-month curves, respectively (**Figure 3D**). **Figure 3E** demonstrates the high expression of NOD2 and the low expression of PLCG1 in the high-risk group. Meanwhile, good prediction results can also be obtained by applying the same risk score formula to PFS analysis (**Supplementary Figure 2**).

A total of 90 patients with GBM from the CGGA cohort (CGGA325) were utilized as the validation set. Based on the same risk score formula and best cutoff value of risk score in the TCGA cohort, patients with GBM in the CGGA cohort were classified into a low-risk and a high-risk group (**Figure 3F**). There were fewer deaths of and longer survival times for the patients in the low-risk subgroup than those in the high-risk subgroup (**Figure 3F**). Furthermore, KM analysis also indicated a significant difference in the survival rate between the low- and high-risk groups (*p* < 0.001, **Figure 3G**). ROC curve analysis of the CGGA cohort showed that our model had good predictive efficacy (AUC = 0.568 for 6-month survival, 0.561 for 12-month survival, and 0.739 for 24-month survival) (**Figure 3H**). The expression of PLCG1 and NOD2 in the high-risk group of GBM was consistent with the TCGA cohort (**Figure 3I**).

**Figure 4:**
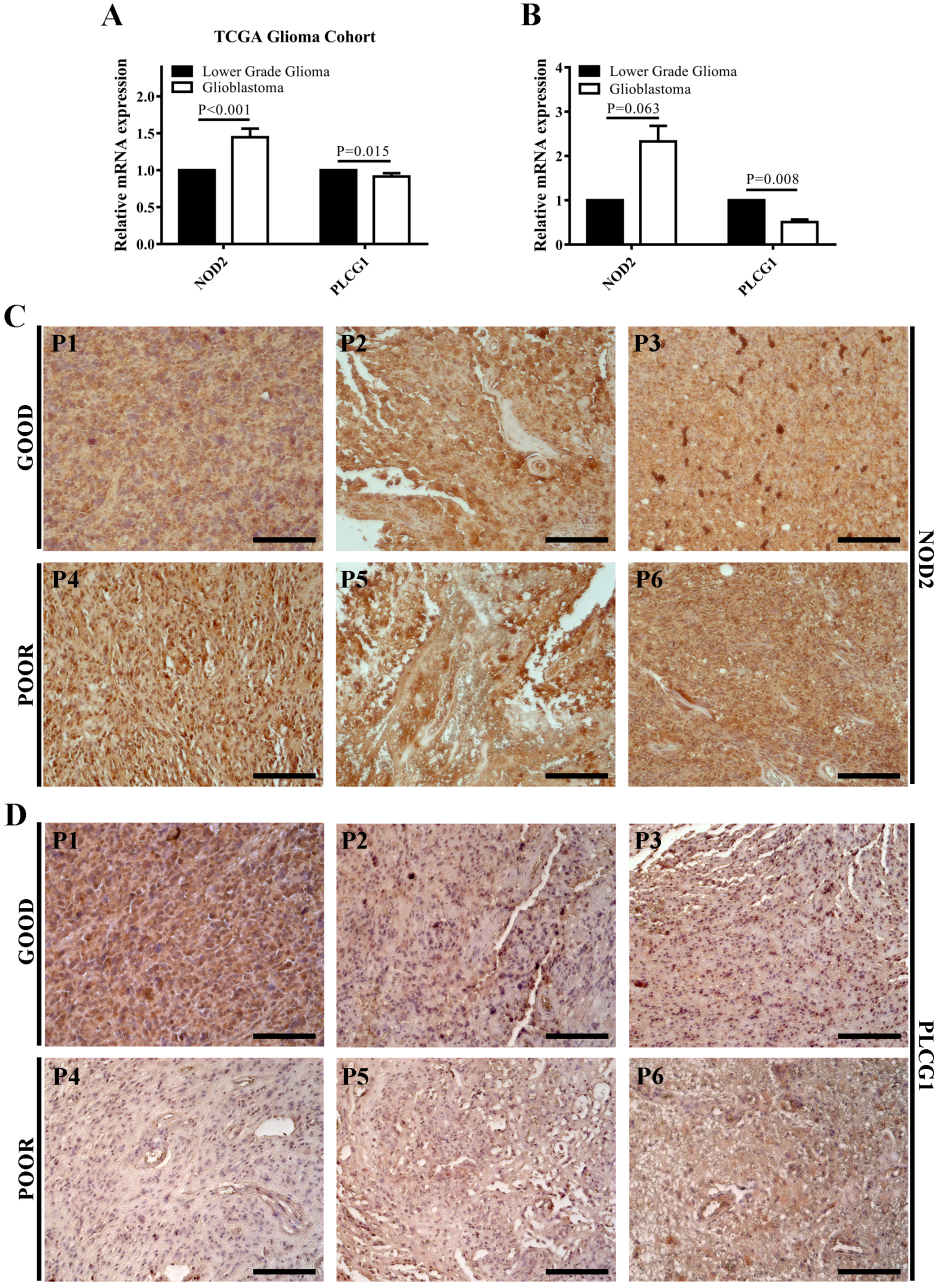
NOD2 and PLCG1 were verified at the mRNA and protein level by experiments. **(A)** The mRNA expressions of NOD2 and PLCG1 in the TCGA glioma cohort. **(B)** The mRNA expressions of NOD2 and PLCG1 in lower-grade glioma (WHO grade 2 and WHO grade 3) and GBM were detected using RT-qPCR. **(C)** Immunohistochemical staining of NOD2 in long-term stable GBM samples (Good, *n* = 3) and rapidly progressing GBM samples (Poor, *n* = 3). **(D)** Immunohistochemical staining of PLCG1 in long-term stable GBM samples (Good, *n* = 3) and rapidly progressing GBM samples (Poor, *n* = 3). Scale bar = 200 μm. P1–P3, long-term stable GBM patients 1–3; P4–P6, rapidly progressing GBM patients 4–6.

TCGA glioma cohort analysis and RT-qPCR results showed that PLCG1 transcription was significantly decreased, while NOD2 transcription was elevated in patients with GBM compared with patients with LGG (**Figures 4A, B**). Immunohistochemical results showed decreased PLCG1 and increased NOD2 in patients with rapidly progressing GBM relative to patients with long-term stable GBM (**Figures 4C, D**). Our experimental results are consistent with those from the public databases above, suggesting that elevated NOD2 expression and reduced PLCG1 expression in GBM represent poor prognosis with a higher risk.

### Independent prognostic value of the risk score

**Figure 5:**
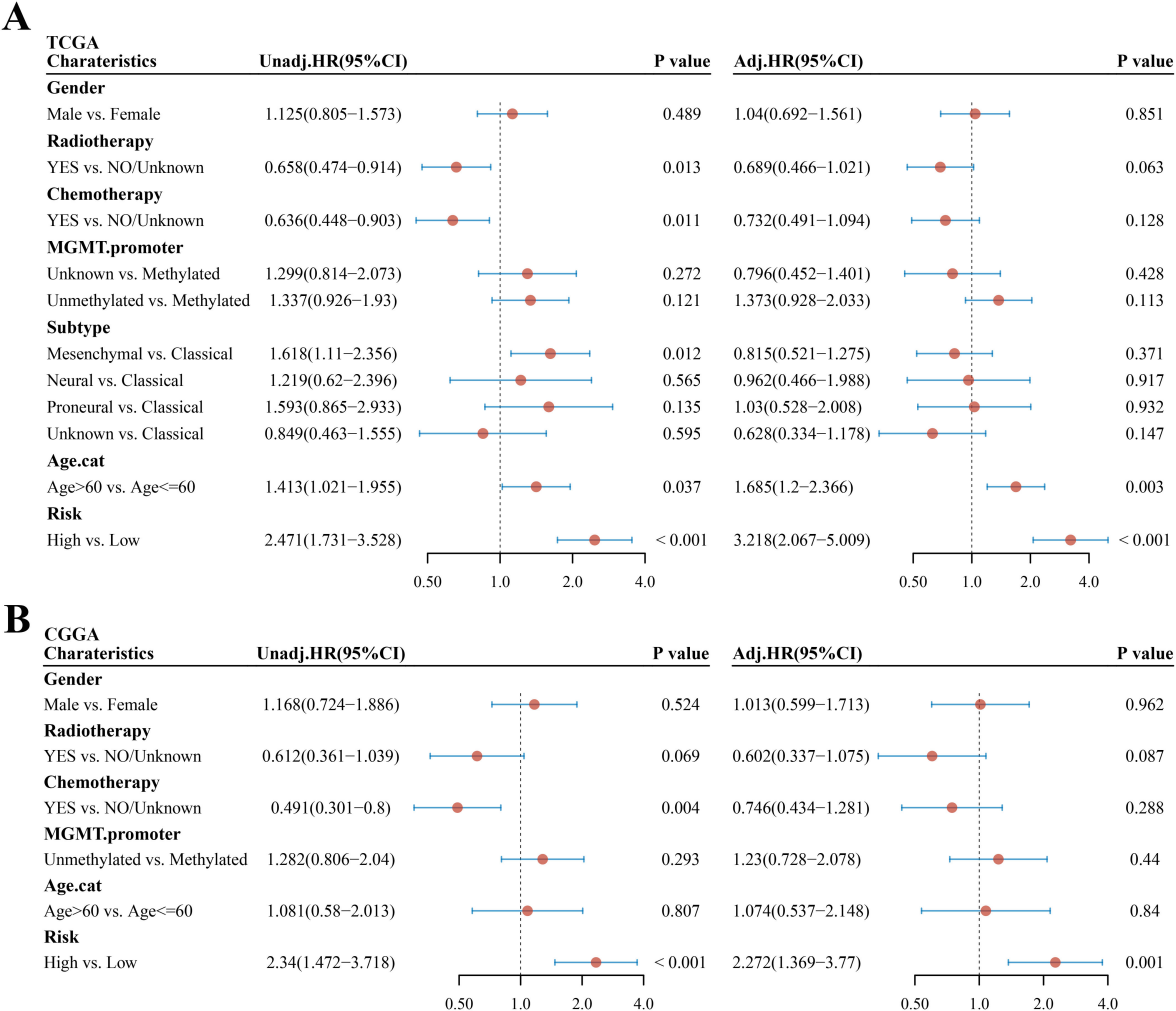
Univariate and multivariate Cox regression analyses in GBM. **(A, B)** Univariate and multivariate analyses of clinical parameters and risk score in the TCGA cohort and CGGA cohort, respectively. Unadj, univariate Cox regression; adj, multivariate Cox regression.

The univariate and multivariate Cox regression analyses were performed to evaluate whether the risk score was an independent prognostic factor of the GBM. The univariate analysis showed that age (HR = 1.413, 95% CI: 1.021–1.955, *p* = 0.037, **Figure 5A**) and risk score (HR = 2.471, 95% CI: 1.731–3.528, *p* < 0.001, **Figure 5A**) were significantly associated with OS. The multivariate Cox regression analysis revealed that, after adjusting for other confounding factors, risk score and age were the independent factors predicting poor survival in the TCGA cohorts (HR = 3.218, 95% CI: 2.067–5.009, *p* < 0.001 and HR = 1.685, 95% CI: 1.2–2.366, *p* = 0.003, **Figure 5A**). To prove that the risk score could serve as an independent prognostic factor, the univariate and multivariate Cox regression analyses were also used in the CGGA cohort and obtained the same result (univariate analysis: HR = 2.34, 95% CI: 1.472–3.718, *p* < 0.001; multivariate analysis: HR = 2.272, 95% CI: 1.369–3.77, *p* = 0.001, **Figure 5B**).

### Building and validating the predictive nomogram

**Figure 6:**
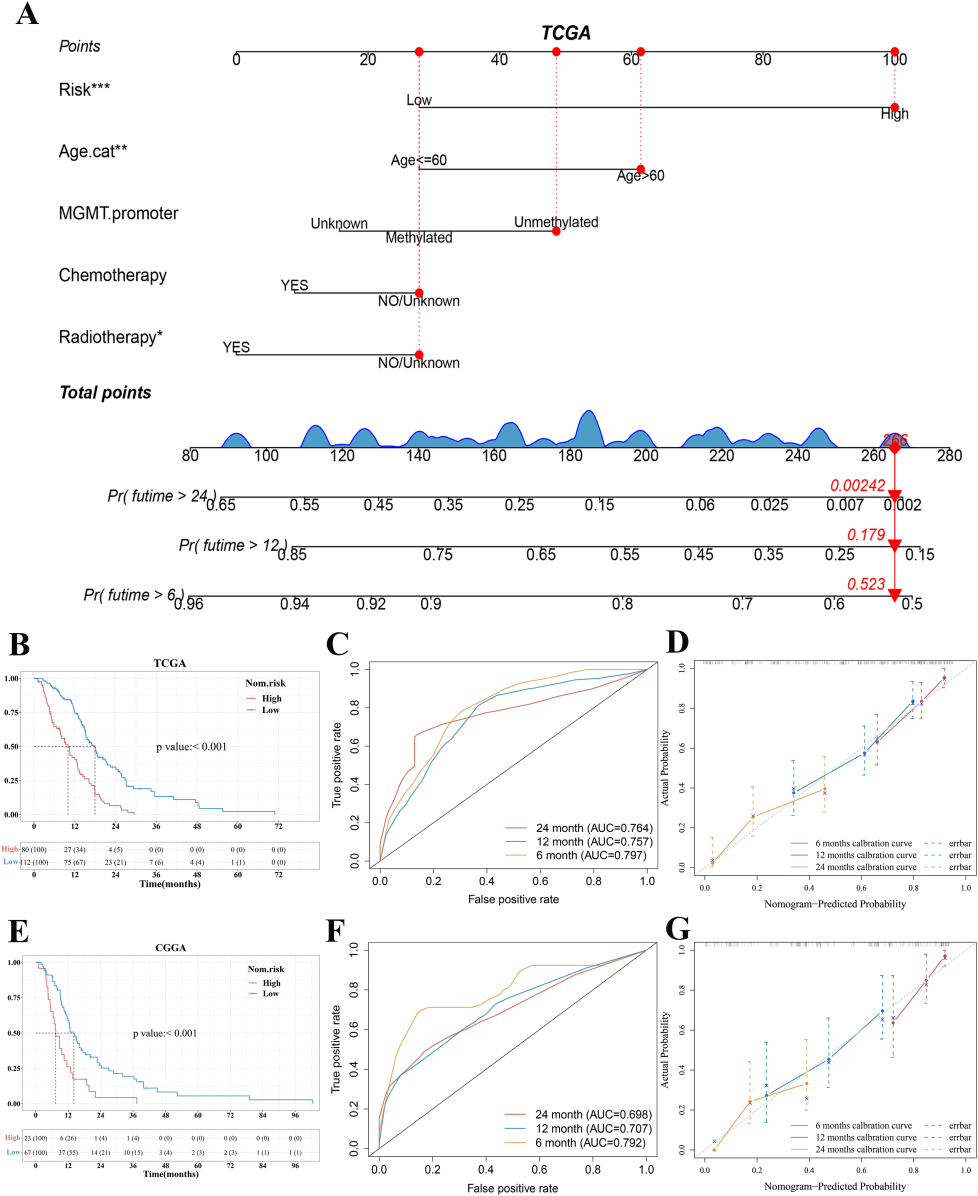
Construction and validation of a predictive nomogram. **(A)** Nomogram for predicting 6-, 12-, and 24-month overall survival for patients with GBM in the TCGA cohort. **(B, E)** Kaplan–Meier curves of patients with GBM in the high-risk and low-risk group based on the nomogram model in the TCGA and CGGA cohort, respectively. **(C, F)** The ROC curve of measuring the predictive value based on the nomogram model in the TCGA and CGGA cohort, respectively. **(D, G)** Calibration curves of nomograms in terms of the agreement between predicted and observed 6-, 12-, and 24-month outcomes in the TCGA cohort and CGGA cohort, respectively. A dashed diagonal line represents the ideal nomogram. ***p < 0.001, **p < 0.01, *p < 0.05. GBM, glioblastoma.

Owing to the importance of the risk score in predicting the prognosis of patients with GBM, we next attempted to explore its value for clinical application. We extracted seven variables, including six clinical features that are generally believed to have a certain impact on the prognosis of GBM (gender, radiotherapy, chemotherapy, MGMT promoter, subtype, and age) and risk score. Then, a nomograph featuring five variables (risk score, age, MGMT promoter, chemotherapy, and radiotherapy) that were selected by stepwise Cox regression was built to predict the survival rates of patients with GBM at 6, 12, and 24 months (**Figure 6A**). Using the median total points of predictive nomogram, patients with GBM were separated into a high- and a low-risk group. We found that the low-risk group had a clear survival advantage over the high-risk group (**Figure 6B**) and further proved that the nomogram model revealed good predictive efficacy for the 6-, 12-, and 24-month survival of patients with GBM (AUC of 0.797, 0.757, and 0.764 in the 6-, 12-, and 24-month ROC curves, respectively, **Figure 6C**).

We further used bootstrap sampling method and external verification method to evaluate the prediction ability of the nomogram. The C-index and Brier score based on the bootstrap sampling method indicated that the nomogram had a good predictive value (**Supplementary Excel Files 3** and **4**). According to the bootstrap sampling method, calibration curves of nomograms suggested that the 6‐, 12-, and 24‐month OS rates could be predicted relatively well compared with an ideal model in the entire cohort (**Figure 6D**). The CGGA cohort was utilized as the external validation set. In the validation set of CGGA325, the results of KM analysis, ROC curve analysis, and calibration curves based on the TCGA cohort nomogram model were consistent with those of the TCGA cohort (**Figures 6E–G**).

### Treatment subgroup analysis, drug sensitivity analysis, and TMB and MIS analysis based on the risk score model in GBM

**Figure 7:**
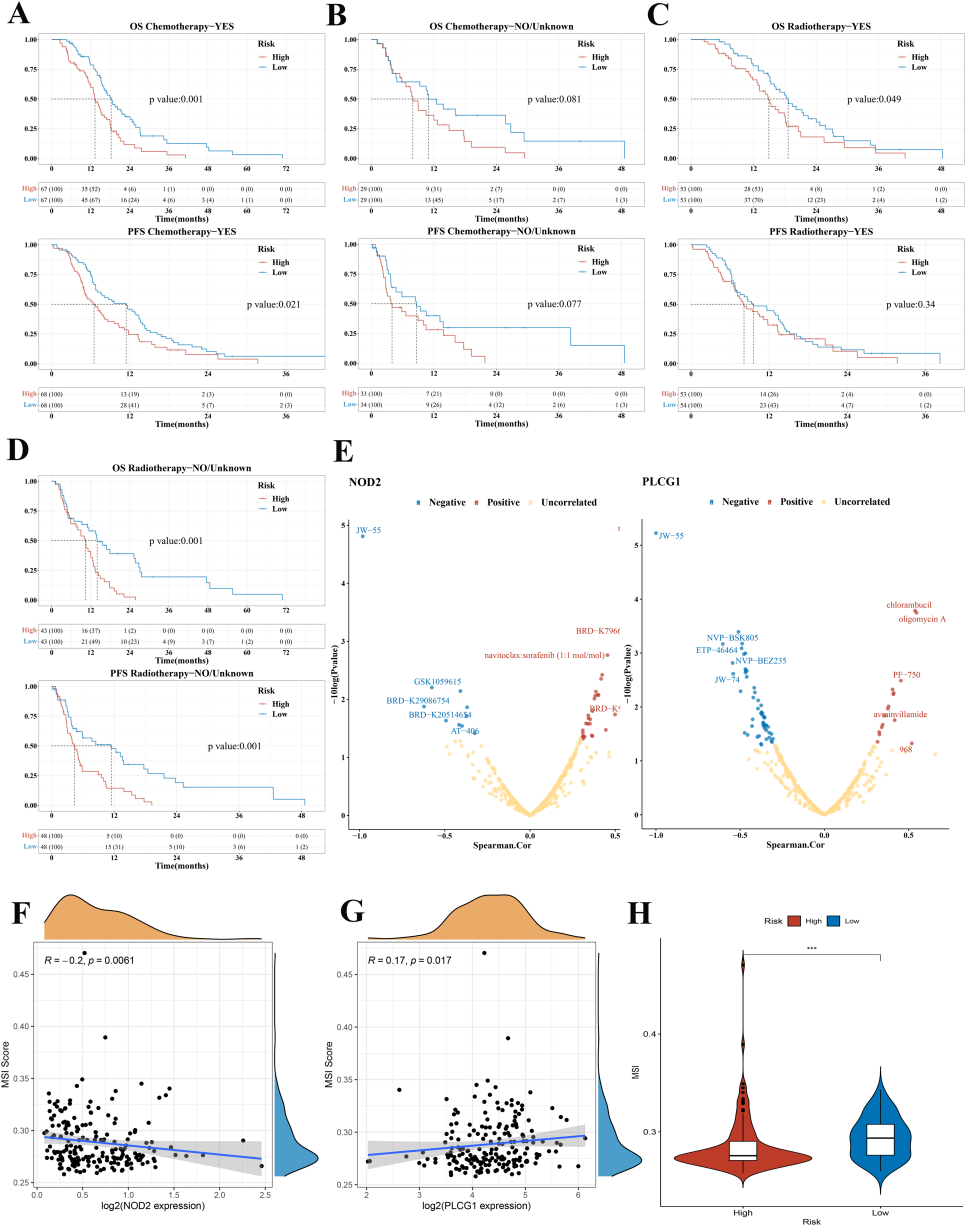
Treatment subgroup analysis, drug sensitivity analysis, and MIS analysis of GBM in the TCGA cohort. **(A)** OS and PFS curves in the high-/low-risk group of patients with GBM who received chemotherapy. **(B)** OS and PFS curves in the high-/low-risk group of patients with GBM who did not receive chemotherapy. **(C)** OS and PFS curves between the low-risk group and the high-risk group in radiotherapy patients. **(D)** OS and PFS curves between the low-risk group and high-risk group in non-radiotherapy patients. **(E)** The correlation between drug sensitivity and the expression of NOD2 and PLCG1 in GBM (positive correlation: Spearman coefficient > 0.3; negative correlation: Spearman coefficient < −0.3). **(F, G)** The correlation between MSI score and NOD2, PLCG1. **(H)** Violin plots comparing the MSI among risk subgroups in the TCGA cohort. OS, overall survival; PFS, progression-free survival; GBM, glioblastoma; MSI, microsatellite instability, ***p < 0.001.

Based on the results of the above analysis, we found that risk score could play an important role in clinical prediction. Next, we investigated whether the risk score model would have a guiding value for GBM clinical treatment. Patients were divided based on whether they received chemotherapy or radiotherapy. The KM plots indicated that the low-risk group had significantly better OS and PFS than the high-risk group in patients who received chemotherapy (median OS 18.2 vs. 13.3 months, *p* = 0.001; median PFS 11.4 vs. 6.5 months, *p* = 0.02, **Figure 7A**). For patients who did not receive chemotherapy, there was no statistical difference in OS and PFS between the low-risk and high-risk groups (median OS 10.9 vs. 7.9 months, *p* = 0.081; median PFS 8.6 vs. 3.9 months, *p* = 0.077, **Figure 7B**). The KM curve showed that the low-risk group had a better OS than the high-risk group in radiotherapy patients (median OS 18.6 vs. 14.9 months, *p* = 0.049, **Figure 7C**). There was no difference in PFS between the low-risk group and the high-risk group in patients who received radiotherapy (median PFS 9.5 vs. 8.1 months, *p* = 0.34, **Figure 7C**). Among non-radiotherapy patients, KM analysis showed that the OS and PFS of patients with GBM in the low-risk group were better than those in the high-risk group (median OS 13.9 vs. 10.4 months, *p* = 0.001; median PFS 11.4 vs. 4.3 months, *p* = 0.001, **Figure 7D**). According to median OS and PFS, we found that the benefit of chemotherapy or radiotherapy was greater in the high-risk group than in the low-risk group. Consistent results were obtained in the validation set (**Supplementary Figures 3A–D**). The treatment subgroup analysis was validated in the CGGA dataset with OS as the endpoint.

To develop a therapy target, it is important to analyze the correlation between gene expression and existing drugs. In our study, drug sensitivity analysis revealed that the expression of PLCG1 and NOD2 was correlated with most drugs in the CTRP and CCLE database (**Figure 7E**). The higher the PLCG1 expression level, the lower the sensitivity of GBM to oligomycin A, chlorambucil, PF-750, avrainvillamide, and 968 and the higher the sensitivity to NVP-BSK805, ETP-46464, NVP-BEZ235, JW-74, and JW-55. The higher the expression level of NOD2, the lower the sensitivity of GBM to navitoclax, BRD-K79669418, navitoclax:sorafenib, BRD-K99006945, and BEC and the higher the sensitivity to AT-406, JW-55, and so on. To clarify whether these genes could also serve as biomarkers for immunotherapy, we then analyzed the correlation between two pyroptosis-related genes and TMB as well as MSI in GBM. The results demonstrated that there was no significant correlation between TMB and NOD2 and PLCG1 (**Supplementary Figures 4A, B**). In MSI analysis, MSI was negatively correlated with NOD2 expression (**Figure 7F, p** = 0.0061) and was positively correlated with the expression of PLCG1 (**Figure 7G, p** = 0.017).

Many studies have revealed that patients with higher TMB (22, 23) and MSI (24), when treated with immune checkpoint blockade therapy, were associated with enhanced responses, long-term survival, and lasting clinical benefits. In our study, we found that there was no significant difference between the low-risk group and the high-risk group in TMB analysis (**Supplementary Figure 4C**), but MSI was higher in the low-risk group, which suggested a better immunotherapy response (**Figure 7H**).

### Comparison of the immune activity between subgroups, and infiltrating immune cell fractions and correlation analysis

**Figure 8:**
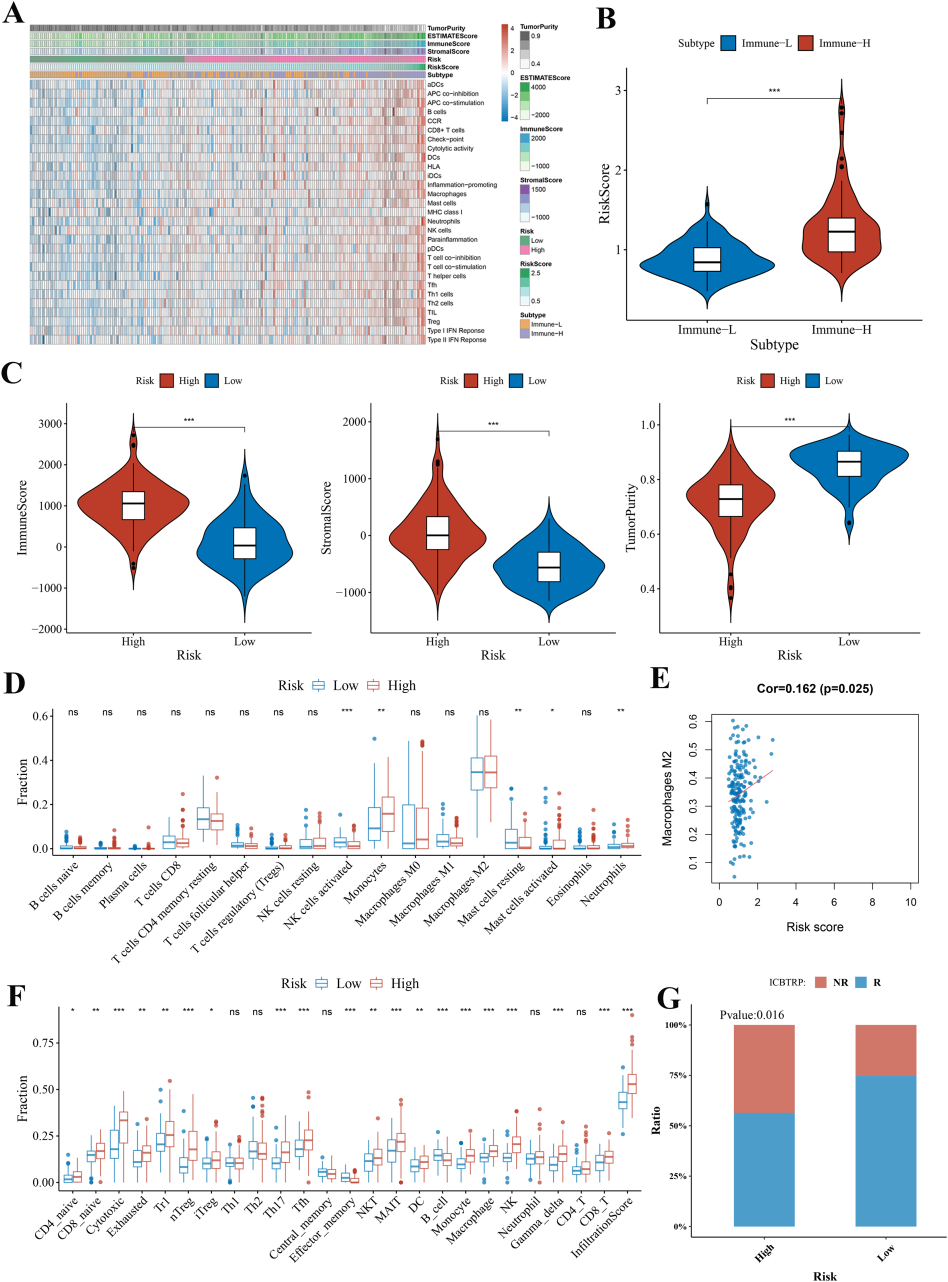
The landscape of immune infiltration levels in the GBM microenvironment and correlation analysis. **(A)** Heatmap showing two immune phenotypes, two risk subgroups, risk score, tumor purity, ESTIMATE, and immune and stromal scores in the GBM microenvironment of samples from the TCGA cohort. **(B)** Violin plot comparing the risk score among immune phenotype in the TCGA cohort. **(C)** Violin plots comparing the immune and stromal scores and tumor purity among risk subgroups in the TCGA cohort. **(D, F)** Immune cell infiltration level of GBM microenvironment among risk subgroups in the TCGA cohort based on the CIBERSORTx algorithm and ImmuCellAI algorithm, respectively. **(E)** The correlation between macrophage M2 and risk score analyzed by CIBERSORTx in the TCGA cohort. **(G)** Bar plot demonstrating the response rate to immune checkpoint blockade therapy in the high-risk and low-risk group. *p < 0.05; **p < 0.01; ***p < 0.001. ns, no statistical significance.

GBM samples with distinct extension of inflammatory cell infiltration were classified into “immune-L” and “immune-H” phenotypes with ssGSEA incorporating 29 types of immune pathways. Four immune scores, two immune phenotypes, two risk subgroups, and risk score were employed in the complex heatmap (**Figure 8A**). The risk score was higher in the immune-H group than in the immune-L group (**Figure 8B**). From the ESTIMATE algorithm, the high-risk group was revealed to have a higher immune score, stromal score, and a lower tumor purity score than the low-risk group (**Figure 8C**). These data indicated that the high-risk group had a stronger immune activity than the low-risk group. Although the high-risk group possessed a large number of activated immune pathways, infiltrating immune cells may play a negative role.

Thus, we used CIBERSORTx and ImmuCellAI algorithms to further quantify the proportion of immune cells. According to the CIBERSORTx algorithm, the low-risk group was revealed to have higher proportions of natural killer (NK) cells activated and mast cells resting and have lower proportions of monocytes and neutrophils (**Figure 8D**). Correlations between immune cell and risk score illustrated that the most negative correlations were found among mast cells resting (**Supplementary Figure 5**). The positive correlation was found between risk score and macrophages M2 (immunosuppressive cells) (**Figure 8E**).

Through the ImmuCellAI algorithm, the high-risk group had a higher proportion of CD4 naïve cells, CD8 naïve cells, cytotoxic cells, exhausted cells, type 1 regulatory T cells (Tr1), natural regulatory T cells (nTreg), inducible regulatory T cells (iTreg), T helper cell 17 (Th17), follicular helper T cells (Tfh), natural killer T (NKT) cells, mucosal-associated invariant T (MAIT) cells, dendritic cells (DCs), monocytes, macrophages, NK cells, and CD8+ T cells and had a lower proportion of B cells and effector memory cells (**Figure 8F**). The cytotoxic cells, NK cells, macrophages, DCs, monocytes, nTreg, NKT cells, Th17, and Tr1 showed significant positive correlations with risk score (**Supplementary Figure 5**). Furthermore, ImmuCellAI can be applied to predict the response of patients with GBM to immune checkpoint blockade therapy. The above result of MIS analysis has shown that the high-risk group had a better immunotherapy response (**Figure 7H**). The analysis of response rates to immune checkpoint blockade therapy also verified this conclusion (**Figure 8G**).

Meanwhile, similar conclusions were drawn in all the above analyses in the CGGA cohort (**Supplementary Figures 6**, **7**).

## Discussion

GBMs are the most common malignant tumors in the central nervous system, and the survival rate is still not satisfactory. Molecular signatures related with distinct clinical outcomes have been delineated in various solid tumors to improve clinical management through the development of personalized medicine (25–27). Given that GBMs were reclassified based on the WHO CNS5 in 2021, there is currently no prognostic model for the new definition of GBM. Thus, it is urgent to explore the gene signature model of the newly defined GBM to indicate prognosis and facilitate clinical treatment.

Pyroptosis is an inflammatory form of programmed cell death, which has been found to play dual roles in tumor development and treatment recently. Pyroptosis elicits an inflammatory response by releasing inflammatory factors that can stimulate the transformation of normal cells into tumor cells to some extent (14). Nonetheless, pyroptosis can inhibit tumor growth, making pyroptosis-related genes the potential prognostic and therapeutic target (15). In many solid tumors, such as ovarian cancer, lung adenocarcinoma, and gastric cancer, the prognostic model based on pyroptosis-related genes has been constructed to predict prognosis (10–12). Multiple studies have confirmed that pyroptosis is universal and holds significant importance in GBM (28–30). So far, the role of pyroptosis-related genes in the newly defined GBM has not been described, and our study will be the first to explore the correlation between pyroptosis-related genes and the prognosis and treatment of the newly defined GBM.

We firstly screened pyroptosis-related genes with significant prognostic value in GBM by univariate Cox regression analysis of OS and found a poor OS in patients with GBM with low AIM2, CASP4, IL1B, NLRC4, NOD2, and PYCARD expression and high PLCG1 and SCAF11 expression. Then, a risk score model was generated via LASSO Cox regression analysis based on these eight pyroptosis-related genes. According to the best cutoff value (0.89) of risk score, the patients with GBM were divided into a low-risk and a high-risk group. Subsequent analysis culminated in several consensuses: (1) the KM curve of the TCGA cohort demonstrated that patients in the high-risk group had more deaths and a shorter survival time than those in the low-risk group; (2) the time-dependent ROC curve analysis of the TCGA cohort showed that the risk score model had good predictive efficacy; (3) the univariate and multivariate Cox regression analyses revealed that the risk score was an independent prognostic factor of the GBM; and (4) the predictive nomogram verified the importance and ability of the risk score in predicting the prognosis of patients with GBM again and its value for clinical application. Meanwhile, the validation sets (CGGA325) obtained consistent results in the above analyses based on the same risk score formula of the TCGA cohort.

It is known that, owing to the shorter survival in patients with GBM, few prognostic models were good predictors of survival. Surprisingly, the prognostic model of pyroptosis-related genes that we developed can well predict the prognosis of GBM. In addition, we calculated a universal risk score model, which could be applied to GBM datasets from different sources. Our risk score model was significantly superior to the existing GBM prognostic model. The prognostic model formula by Cheng et al. (31) was only applicable to one dataset, and different risk score formulas need to be developed for different datasets. Nevertheless, the same risk score formula was used for the analysis of the training set and the validation set in our study. The universal prognostic model was very beneficial for clinical application. For example, when prognostic analysis is performed on a clinical patient with GBM, our risk score formula can be used as long as the patient with GBM has the corresponding data, and there is no need to reconstruct the coefficient of this patient prognostic model formula. In conclusion, our prognostic model is conducive to clinical expansion of predicting individualized survival time and optimizing therapeutic approaches for patients with GBM.

In our study, a risk score model featuring two pyroptosis-related genes (PLCG1 and NOD2) was constructed. PLCG1 is involved in the receptor tyrosine kinase (RTK)-mediated signal transduction pathway, thus affecting cell growth, differentiation, and apoptosis (32). Recently, some research (33, 34) demonstrated that PLCG1 could mediate the activity of GSDMD and pyroptosis. We found that low PLCG1 expression was associated with poor survival outcomes, which may function as a tumor suppressor gene in GBM. NOD2 belongs to the Nod-like receptor (NLR) family of innate immune proteins that play fundamental and pleiotropic roles in host defense against infection and in the control of inflammation (35). Shi et al. (36) revealed that the histone H3 could cause pyroptosis through NOD2 pathways. However, the relationships between NOD2-mediated pyroptosis and tumor development remain unknown. We found that the high expression of NOD2 predicted poor survival rates, indicating that it functioned as a tumor-promoting gene in this study. In addition, the results of RT-qPCR and immunohistochemistry showed that elevated NOD2 expression and reduced PLCG1 expression in GBM denote poor prognosis with a higher risk. The experimental results are consistent with those from the risk score model based on public databases.

Despite the tremendous effort that has been devoted to developing novel cancer therapies, the treatment of GBM has remained relatively unchanged for decades and consists of surgical resection followed by adjuvant chemoradiotherapy (37). There is growing evidence that the identification of prognostic factors is important for the optimal treatment of patients with GBM (38, 39). Therefore, we further explored whether risk score model would have a guiding value for GBM clinical therapeutic management. Treatment subgroup analysis concluded that patients in the low-risk group were found to have longer survival times than those in the high-risk group regardless of whether they received chemotherapy or radiotherapy. However, the benefit of chemotherapy or radiotherapy was greater in the high-risk group than in the low-risk group according to median OS and PFS. Recently, the remarkable research progress of targeted therapy and immunotherapy among diverse cancer types has brought a promising prospect for exploring the novel treatment of GBM. To develop the GBM therapy target, we analyzed the correlation between existing drugs and the two genes included in the risk score model. Drug sensitivity analysis revealed that the expression of PLCG1 and NOD2 was correlated with most drugs’ sensitivity; for example, the higher the expression of PLCG1 and NOD2, the higher the sensitivity of GBM to JW-55 (an effective selective β-catenin inhibitor). To clarify whether the risk score model could guide immunotherapy for patients with GBM, TMB and MSI analyses were performed and the results revealed that the low-risk group had a better immunotherapy response. All in all, the risk score model had significant value for guiding the clinical treatment of patients with GBM.

The GBM microenvironment consists of not only tumor cells but also stromal cells, including distinct immune cell subsets (40). Tumor-infiltrating immune cells and stromal cells are associated with angiogenesis, immune suppression, chemotherapeutic resistance, and tumor cell migration (41). In order to explore the correlation between the risk score and immune infiltration level of GBM, the ESTIMATE algorithm and ssGSEA were used for analysis, and the results indicated that the high-risk group had stronger immune activity than the low-risk group. However, the survival of patients in the high-risk group was shorter. The inconsistent results may be related to the negative role of infiltrating immune cells. Then, the CIBERSORTx analysis described the immune infiltrating cells were mainly macrophages M2 (immunosuppressive cells) in the GBM patients regardless of low-risk or high-risk group. The ImmuCellAI analysis, as a supplement to CIBERSORTx analysis, focused on the infiltration of various T cells. Although there were a variety of immune activation-related cells that infiltrated in the high-risk group, such as NK cells, DCs, and CD8 T cells, the level of these cells was low.

Based on these findings, the poor survival outcome of high-risk GBMs may be caused by decreased levels of antitumor immunity. Furthermore, ImmuCellAI analysis also verified that the low-risk group had a better immune checkpoint blockade therapy response. To sum up, immune infiltration data demonstrated that the correlations between the risk score and the tumor immune microenvironment are important for immunotherapy.

Our study aimed to investigate the prognostic value of pyroptosis-related genes, build a prognostic model, verify its value of guiding clinical treatment in the newly defined GBM, and explore the relationship between the prognostic model of pyroptosis-related genes and the tumor immune microenvironment. Although we had performed multi-angle and multi-database verifications, this study still had limitations that need to be considered. Firstly, because of the lack of sufficient molecular characteristics of LGG in the CGGA database, it is impossible to determine whether adult diffuse astrocytic tumors that were IDH-wildtype with TERT promoter mutation or EGFR gene amplification or +7/−10 chromosome copy number alterations. Therefore, this LGG type cannot be reclassified as GBM in the validation set. Secondly, although our research methodology resembled that of Chao et al. (28), the present study applies this approach to the newly defined GBM entity under the WHO CNS5 (2021) classification. Furthermore, we implemented more stringent cohort selection criteria specifically designed for this reclassified population, contrasting with studies involving broader glioma cohorts.

In summary, our study provides strong evidence for the prognosis and clinical management of the newly defined GBM. First, the risk score model included two pyroptosis-related genes that hold important prognostic value for patients with GBM. In particular, our experimental results are consistent with those from the risk score model, indicating that elevated NOD2 expression and reduced PLCG1 expression in GBM represent poor prognosis with a higher risk. Second, the risk score model guides not only traditional chemoradiotherapy but also novel therapies, such as targeted therapy and immunotherapy. Meanwhile, immune infiltration analysis offers a significant basis for future studies of the relationships between pyroptosis-related genes and immunity in GBM. To the best of our knowledge, this is the first study to conduct bioinformatics and experimental analysis on the newly defined GBM.

## Data Availability

The original contributions presented in the study are included in the article/**Supplementary Material**. Further inquiries can be directed to the corresponding authors.
