# Supplementary material for: The pyroptosis-related gene signature predicts prognosis and reveals immune microenvironment infiltration in reclassified glioblastoma based on 2021 WHO classification
Source: Front Immunol. 2025 Jul 21;16:1617036. doi: 10.3389/fimmu.2025.1617036 (PMC12318951; doi:10.3389/fimmu.2025.1617036)
Supplement: Supplementary file 1 [file DataSheet1.zip › Supplementary Table 1.docx]

| **Variables** | **Total (n = 282)** | **CGGA (n = 90)** | **TCGA (n = 192)** | **p** |
| --- | --- | --- | --- | --- |
| **Overall survival (days), Median (Q1,Q3)** | 372 (212.5, 550) | 346.5 (218.25, 585) | 383 (204.75, 545) | 0.784 |
| **State, n (%)** |  |  |  | 0.015 |
| Dead | 229 (81) | 81 (90) | 148 (77) |  |
| Live | 53 (19) | 9 (10) | 44 (23) |  |
| **Gender, n (%)** |  |  |  | 0.486 |
| Female | 107 (38) | 31 (34) | 76 (40) |  |
| Male | 175 (62) | 59 (66) | 116 (60) |  |
| **Age, Mean ± SD** | 57.25 ± 12.11 | 50.73 ± 11.19 | 60.31 ± 11.31 | < 0.001 |
| **Radiotherapy, n (%)** |  |  |  | < 0.001 |
| NO/Unknown | 106 (38) | 20 (22) | 86 (45) |  |
| YES | 176 (62) | 70 (78) | 106 (55) |  |
| **Chemotherapy, n (%)** |  |  |  | 0.782 |
| NO/Unknown | 83 (29) | 25 (28) | 58 (30) |  |
| YES | 199 (71) | 65 (72) | 134 (70) |  |
| **MGMT.promoter, n (%)** |  |  |  | < 0.001 |
| Methylated | 100 (35) | 31 (34) | 69 (36) |  |
| Unknown | 28 (10) | 0 (0) | 28 (15) |  |
| Unmethylated | 154 (55) | 59 (66) | 95 (49) |  |
| **Subtype, n (%)** |  |  |  | < 0.001 |
| Classical | 74 (26) | 0 (0) | 74 (39) |  |
| Mesenchymal | 70 (25) | 0 (0) | 70 (36) |  |
| Neural | 13 (5) | 0 (0) | 13 (7) |  |
| Proneural | 14 (5) | 0 (0) | 14 (7) |  |
| Unknown | 111 (39) | 90 (100) | 21 (11) |  |
| **Age.cat, n (%)** |  |  |  | < 0.001 |
| Age<=60 | 175 (62) | 77 (86) | 98 (51) |  |
| Age>60 | 107 (38) | 13 (14) | 94 (49) |  |
