# Supplementary material for: The pyroptosis-related gene signature predicts prognosis and reveals immune microenvironment infiltration in reclassified glioblastoma based on 2021 WHO classification
Source: Front Immunol. 2025 Jul 21;16:1617036. doi: 10.3389/fimmu.2025.1617036 (PMC12318951; doi:10.3389/fimmu.2025.1617036)
Supplement: Supplementary file 1 [file DataSheet1.zip › supplementary legends.docx]

**Supplementary materials legends**

**Supplementary Figure 1** The prognostic value of pyroptosis-related gene in newly defined GBM. **(A)** The overall survival curve of AIM2, CASP4, IL1B, PYCARD, NOD2, NLRC4, PLCG1 and SCAF11 in GBM patients in the high-/low-expression group. **(B)** The progression free survival curve of AIM2, CASP4, GPX4, GSDMC, IL1B, NLRC4, NLRP3, NOD2, PYCARD and PLCG1 in the high-/low-expression group. GBM: glioblastoma.

**Supplementary Figure 2** Construction of a prognostic pyroptosis-related gene model based on PFS in the TCGA cohort. **(A)** Distribution of risk score in GBM patients. **(B)** The expression of PLCG1 and NOD2 in GBM. **(C)** Kaplan–Meier curves for the PFS in the high- and low-risk groups. **(D)** The survival status for each patient (low-risk

population: on the left side of the dotted line; high-risk population: on the right side of the dotted line). GBM: glioblastoma.

**Supplementary Figure 3** Treatment subgroup analysis of GBM in CGGA cohort. **(A)** OS curves in the high-/low-risk group of GBM patients that received chemotherapy. **(B)** OS in the high-/low-risk group of GBM patients who don’t received chemotherapy. **(C)** OS curves between the low-risk group and high-risk group in non-radiotherapy patients. **(D)** OS curves between the low-risk group and high-risk group in radiotherapy patients.

**Supplementary Figure 4** TMB analysis of GBM in TCGA cohort. **(A, B)** The correlation between TMB score and NOD2, PLCG1. **(C)** Violin plots comparing the MSI among risk subgroups in the TCGA cohort.

**Supplementary Figure 5** The correlation between immune infiltrating cells and risk score analyzed, respectively, by ImmuCellAI **(A-I)** and CIBERSORTx **(J)** in TCGA cohort. |Cor| > 0.3 and P <0.05 were considered to be significantly correlated.

**Supplementary Figure 6** The landscape of immune infiltration levels in the GBM microenvironment and correlations analysis. **(A)** Heatmap showing two immune phenotypes, two risk subgroups, risk score, tumor purity, ESTIMATE, immune and stromal scores in the GBM microenvironment of samples from the CGGA cohort. **(B)** Violin plot comparing the risk score among immune phenotype in the CGGA cohort. **(C)** Violin plots comparing the immune and stromal scores and tumor purity among risk subgroups in the TGGA cohort. **(D, F)** Immune cell infiltration level of GBM microenvironment among risk subgroups in the CGGA cohort based on the CIBERSORTx algorithm and ImmuCellAI algorithm, respectively. **(E)** The correlation between mast cells resting and risk score analyzed by CIBERSORTx in CGGA cohort. **(G)** Bar plot demonstrating the response rate to immune checkpoint blockade therapy in the high-risk and low-risk group. *P < 0.05; **P < 0.01; ***P < 0.001.

**Supplementary Figure 7** The correlation between immune infiltrating cells and risk score analyzed, respectively, by CIBERSORTx **(A)** and ImmuCellAI **(B)** in CGGA cohort. |Cor| > 0.3 and P <0.05 were considered to be significantly correlated.

**Supplementary Table 1** The baseline characteristics of newly defined GBM in the TCGA cohort and CGGA cohort.

**Supplementary Text file** Detailed usage of the R packages in our analysis.

**Supplementary Excel file 1** The 33 pyroptosis-related genes.

**Supplementary Excel file 2** The related information on risk-score model.

**Supplementary Excel file 3** The C-index and Brier Score based on bootstrap or non-bootstrap sampling method at 6-, 12- and 24-month survival.

**Supplementary Excel file 4** The C-index Score based on bootstrap or non-bootstrap sampling method at each survival time.
