# Supplementary figures and images for: The pyroptosis-related gene signature predicts prognosis and reveals immune microenvironment infiltration in reclassified glioblastoma based on 2021 WHO classification
Source: Front Immunol. 2025 Jul 21;16:1617036. doi: 10.3389/fimmu.2025.1617036 (PMC12318951; doi:10.3389/fimmu.2025.1617036)

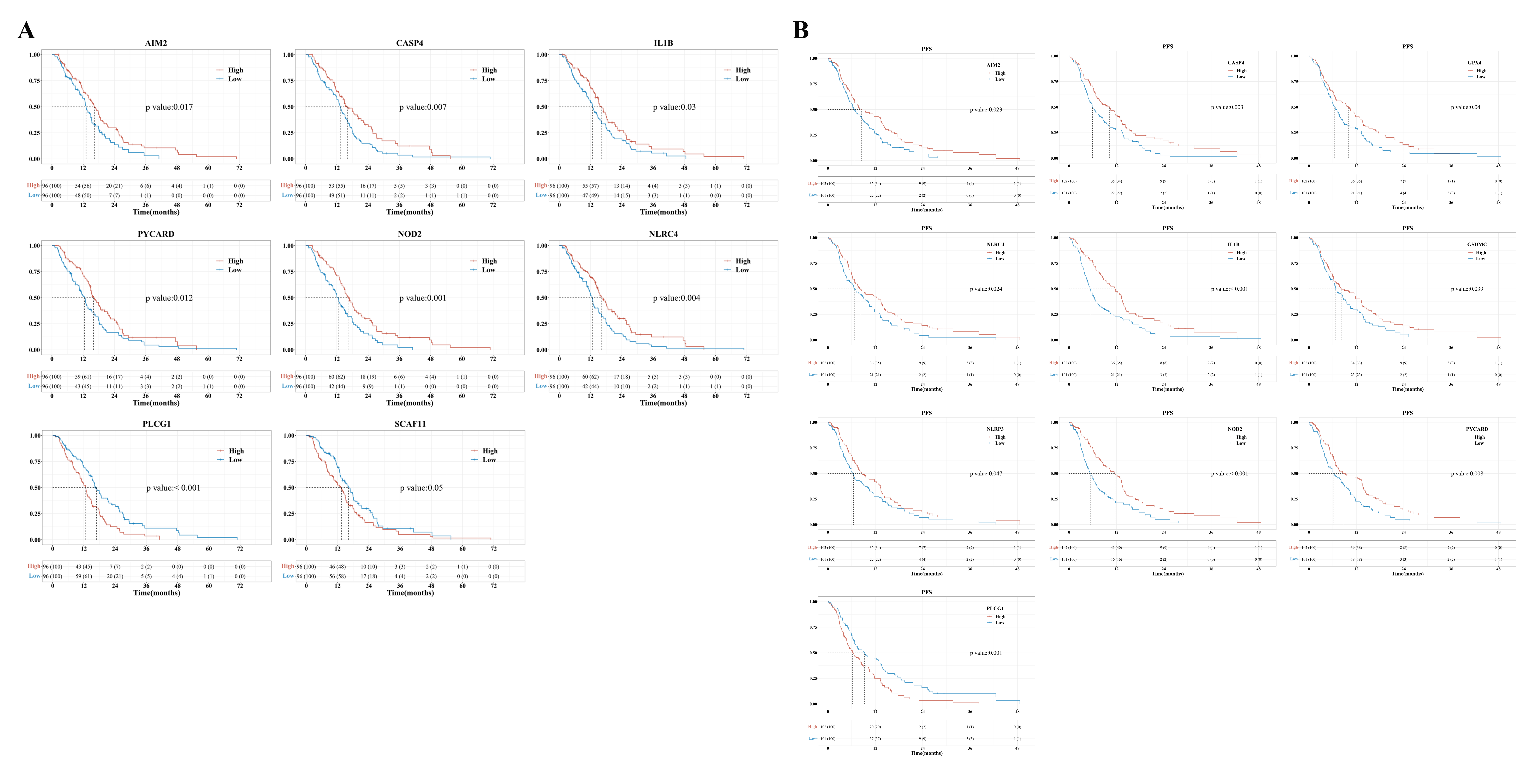

Supplement: Supplementary file 1 [file DataSheet1.zip › Supplementary Fig. 1.tif]

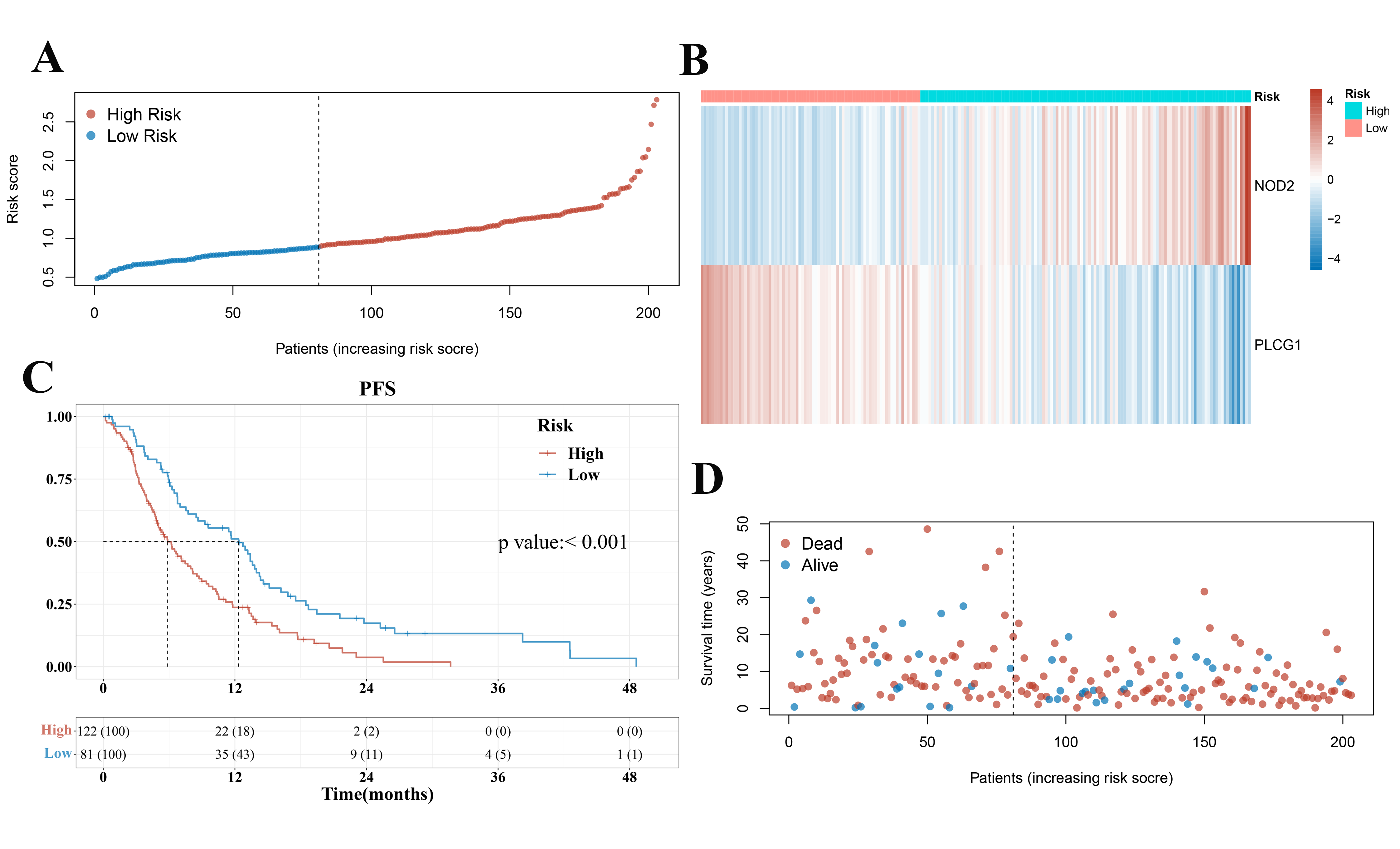

Supplement: Supplementary file 1 [file DataSheet1.zip › Supplementary Fig. 2.tif]

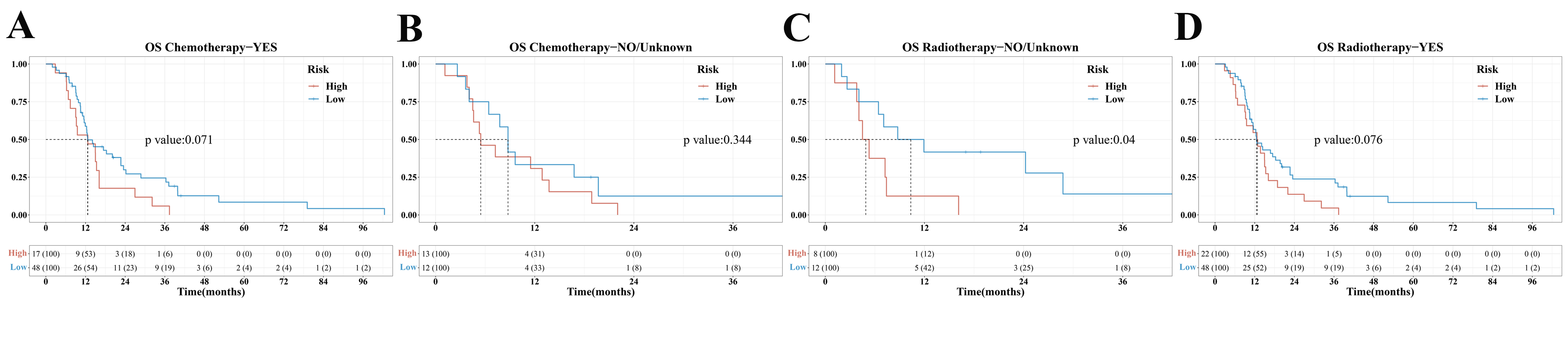

Supplement: Supplementary file 1 [file DataSheet1.zip › Supplementary Fig. 3.tif]

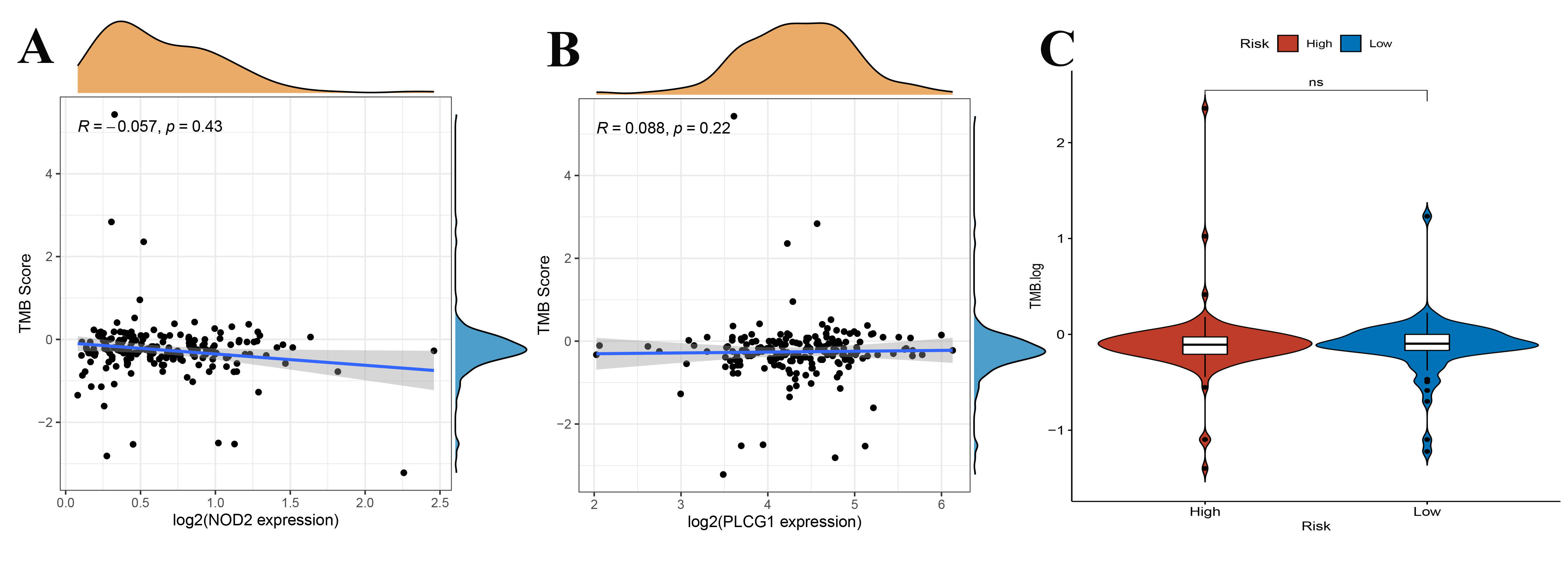

Supplement: Supplementary file 1 [file DataSheet1.zip › Supplementary Fig. 4.tif]

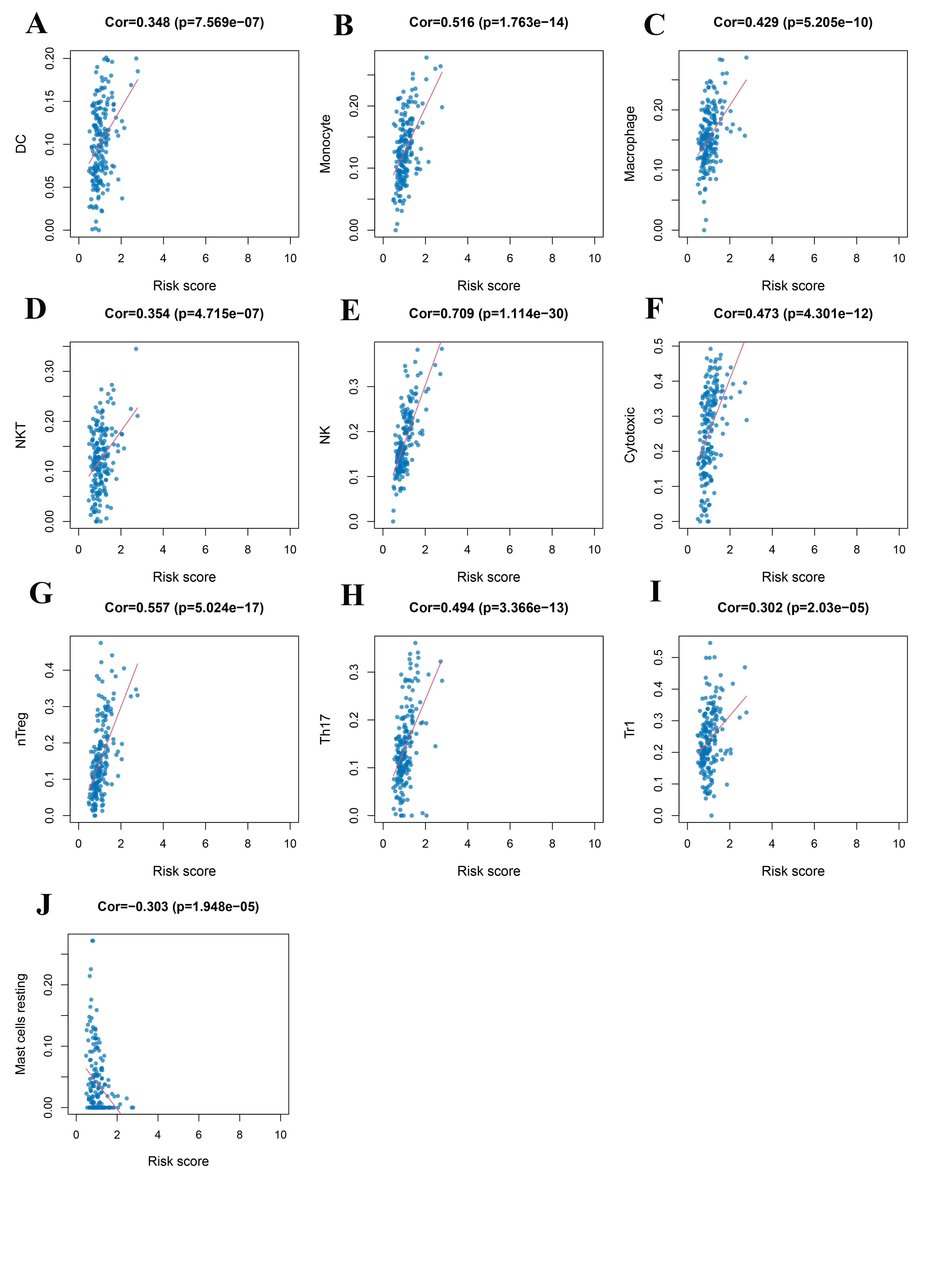

Supplement: Supplementary file 1 [file DataSheet1.zip › Supplementary Fig. 5.tif]

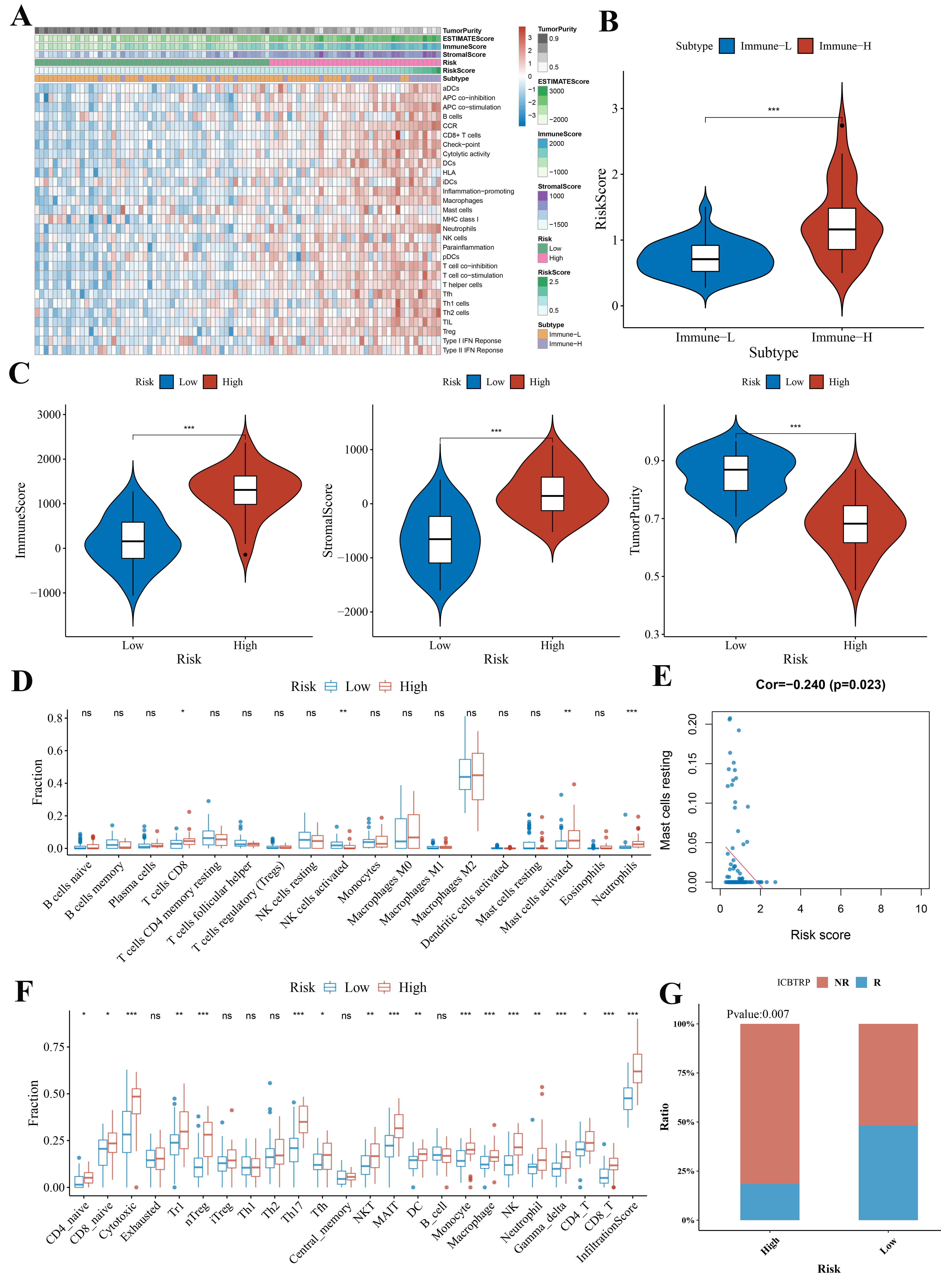

Supplement: Supplementary file 1 [file DataSheet1.zip › Supplementary Fig. 6.tif]

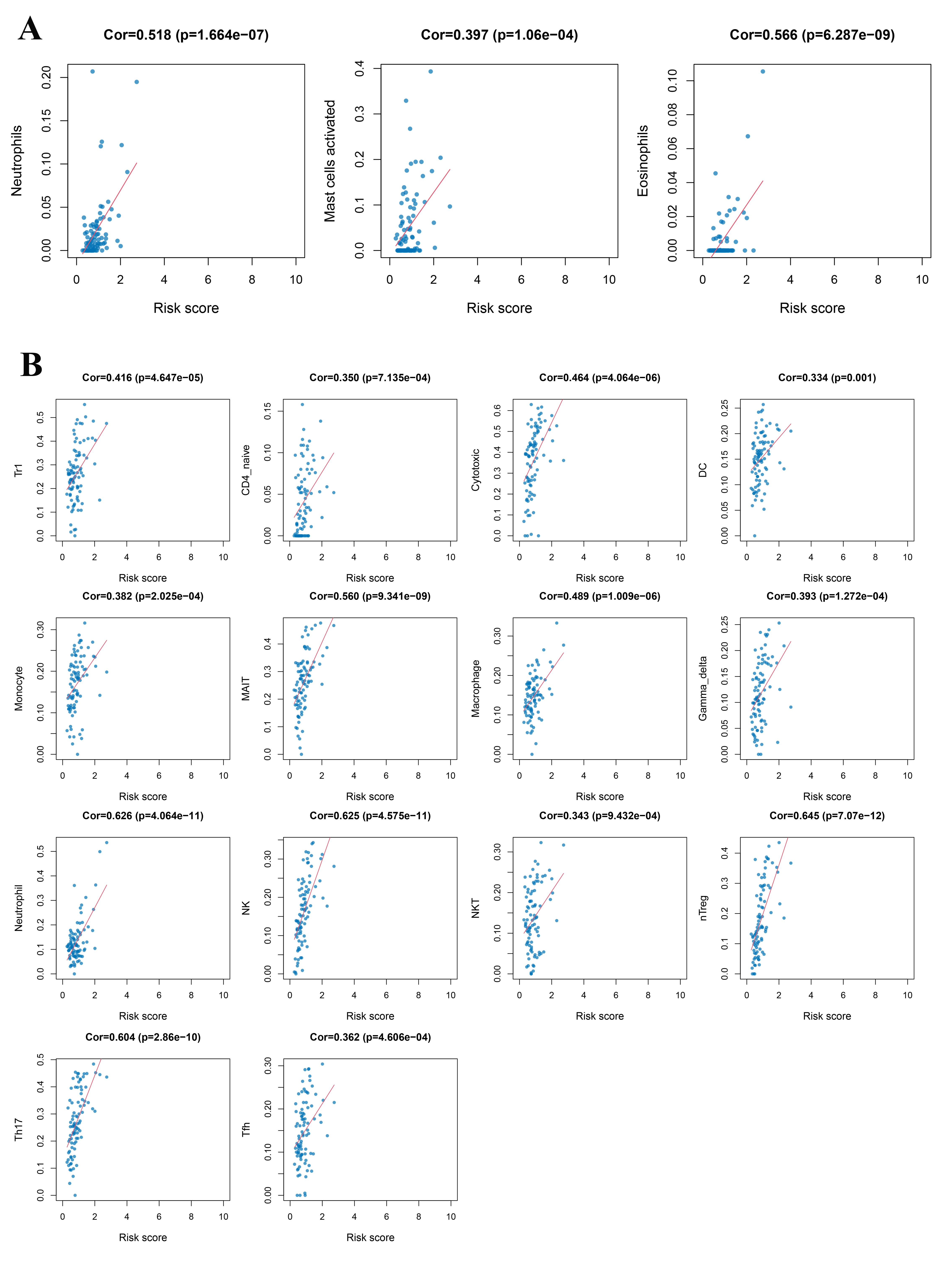

Supplement: Supplementary file 1 [file DataSheet1.zip › Supplementary Fig. 7.tif]
